# Novel exon mutation in *SYCE1* gene is associated with non‐obstructive azoospermia

**DOI:** 10.1111/jcmm.17180

**Published:** 2022-01-12

**Authors:** Ke Feng, Hengtao Ge, Huanhuan Chen, Chenchen Cui, Shan Zhang, Cuilian Zhang, Li Meng, Haibin Guo, Lei Zhang

**Affiliations:** ^1^ Reproductive Medicine Center Henan Provincial People’s Hospital People's Hospital of Zhengzhou University Henan Provincial People’s Hospital of Henan University Zhengzhou China; ^2^ Henan Joint International Research Laboratory of Reproductive Bioengineering Zhengzhou China; ^3^ Reproductive Medicine Center Henan Provincial People's Hospital Affiliated to Xinxiang Medical College Zhengzhou China

**Keywords:** assisted reproduction, male infertility, NOA, *SYCE1*

## Abstract

Non‐obstructive azoospermia (NOA) is a common cause of male infertility, and genetic problems, such as chromosomal abnormalities and gene mutations, are important causes of NOA. Our centre received a case of NOA, in which no mature sperm was found during microdissection testicular sperm extraction. A postoperative pathological examination revealed that testicular spermatogenesis was blocked. Target region capture combined with high‐throughput sequencing was used to screen for male infertility‐related gene mutations. Sanger sequencing further confirmed that the *SYCE1* gene, a central component of the synaptonemal complex (SC) during meiosis, had a homozygous deletion mutation in the tenth exon (c.689_690del; p.F230fs). Through molecular biological studies, we discovered altered expression and nuclear localization of the endogenous mutant SYCE1. To verify the effects in vitro, wild‐ and mutated‐type *SYCE1* vectors were constructed and transfected into a human cell line. The results showed that the expression and molecular weight were decreased for SYCE1 containing c.689_690del. In addition, mutated SYCE1 was abnormally located in the cytoplasm rather than in the nucleus. In summary, our research suggests that the novel homozygous mutation (c.689_690del; p.F230fs) altered the SYCE1 expression pattern and may have disturbed SC assembly, leading to male infertility and to a barrier to gamete formation. We reported for the first time that a frameshift mutation occurred in the exon region of *SYCE1* in an NOA patient. This study is beneficial for accurate NOA diagnosis and the development of corresponding gene therapy strategies.

## 1 INTRODUCTION

The incidence of infertility is approximately 10%–15%,^1^ and the male factor accounts for nearly half of it.^2^, ^3^ Approximately 15% of male infertility patients have azoospermia,^4^ and azoospermia includes obstructive and non‐obstructive azoospermia (NOA). Obstructive azoospermia (OA) is caused by blockage of the vas deferens in any part, and the causes include serious infection, iatrogenic injury, or congenital abnormalities. NOA accounts for approximately 60% of azoospermia patients^5^; due to abnormal spermatogenesis of the testis, mature sperm cannot be produced.

Approximately 15% of male infertility patients and 25% of azoospermia patients have genetic defects.^6^, ^7^, ^8^, ^9^ The most important genetic factors for azoospermia include *CFTR* gene mutation, aneuploidy and Y chromosome microdeletion.^6^, ^10^, ^11^ In addition, copy number variation (CNV) and mutations in several genes are also causes of azoospermia.^12^ Gene mutations known to affect sperm production and cause NOA include *USP26*, *DAZL*, *MTHFR*, *INSL3*, *AR*, *StAR*, *SYCP3*, *SYCP2*, *SYCE1*, *XRCC2*, *DMC1*, *TAF4B* and *ZMYND15*..^8^, ^12^, ^13^, ^14^, ^15^, ^16^, ^17^ Among them, three genes, namely *SYCP3*, *SYCP2* and *SYCE1*, are related to meiosis. Meiosis is a special method of dividing for sexual reproduction organisms to produce haploid gametes. In the early stage of the first meiosis, a synaptonemal complex (SC) is formed between homologous chromosomes, which guarantees the correct identification, pairing, recombination and separation of homologous chromosomes. SYCP3, SYCP2 and SYCE1 proteins are the main components of SCs and are exceedingly important for the association of homologous chromosomes.^5^, ^13^, ^14^

Human infertility is related to abnormal SC function. *SYCP3* and *SYCP2* are mostly heterozygous mutations with an autosomal dominant inheritance pattern, whereas *SYCE1* is a homozygous pathogenic mutation with a recessive inheritance pattern.^5^, ^13^, ^14^ At present, there are three reports of *SYCE1* mutations in reproductive clinics. One case was a nonsense homozygous mutation leading to primary ovarian insufficiency (POI), which occurred in two sisters in an Israeli Arab family.^18^ The other two cases were homozygous mutations at the splice site, resulting in NOA, both of which occurred in Iranian consanguinity families.^5^, ^19^ *SYCE1* knockout in mice resulted in complete infertility, meiosis block, barrier to SC formation and abnormal double‐strand break repair.^20^ The human *SYCE1* gene is located on chromosome 10q26.3, contains 14 exons, encodes 318 amino acids and is essential for meiosis. The structural core of SYCE1 is located at amino acids 25–179 at the N‐terminus, which mediates the formation of dimers of SYCE1 protein, and its C‐terminus is in a long extended state.^21^ The recruitment of SYCE1 to SCs depends on SYCP1, SYCE3 and SIX6OS1.^22^, ^23^, ^24^ The interaction between SYCE1 and SIX6OS1 was abnormal in patients with NOA and POI caused by the SYCE1 mutation.^25^

In this study, we reported for the first time that a nonsense homozygous mutation (c.689_690del; p.F230fs) occurred in the *SYCE1* exon region of an NOA patient. Our studies confirmed that this new mutation led to abnormal expression and localization of SYCE1 and might affect its involvement in the formation of SCs during meiosis.

## 2 MATERIALS AND METHODS

### 2.1 Patient

The patient, Han nationality, came to the reproductive medicine centre of Henan Provincial People's Hospital for treatment due to years of infertility. The patient's testicular tissue was obtained by puncture, and blood samples from the patient and his parents were obtained for investigation. This study was approved by the Ethics Committee of the Henan Provincial People's Hospital. The patient and related family members were informed of the use of their tissue materials for research purposes and signed a consent form.

### 2.2 Reagents

All chemicals and reagents were purchased from Sigma‐Aldrich, unless otherwise specified.

### 2.3 Target area capture/high‐throughput sequencing strategy

To explore the possible genetic mutations, we used anticoagulant tubes to collect peripheral blood from the NOA patient and his parents. The strategy of target area capture combined with high‐throughput sequencing was adopted to screen male infertility‐related gene mutations and was conducted by a commercial company (Jiabao Medical Laboratory). Briefly, it included the following procedures: (1) DNA extraction, purification and quality inspection of samples; (2) the DNA was broken into 200 bp fragments by ultrasonic interruption and purified; (3) the purified DNA library was constructed; (4) the capture probe system was hybridized with the DNA library to capture the target region; (5) after amplification and enrichment, the captured products were sequenced in PE150 mode on the Illumina MiSeq platform; and (6) bioinformatics analysis of sequencing data. This detection method covered 56 male infertility‐related genes. Detailed information regarding the genes in each of the above categories is presented in the supplementary file (Table S1). The mutation types detected by target area capture combined with high‐throughput sequencing methods include point mutations, small indels (≤10 bp) and homozygous deletions at the exon level. To ensure the accuracy of the data analysis, variations in low coverage in the target area were filtered out. Due to the presence of high‐repetition and low‐complexity regions or pseudogenes in certain genes, the detection cannot completely cover all exon regions; however, the overall coverage can reach >95%.

In silico analysis of the identified mutation was conducted to predict its pathogenicity. For the interpretation and evidence grading of gene variation, the latest version of the American Society of Medical Genetics and Genomics (ACMG) Standards and Guidelines for the Interpretation of Sequence Variants was implemented (PVS1+PM2‐supporting + PM3‐supporting), and the reference genome was hg19.

### 2.4 Sanger sequencing

In this study, the classic Sanger sequencing method was used to verify the *SYCE1* mutation in the patient found by high‐throughput sequencing, and to detect the *SYCE1* gene DNA sequence in the peripheral blood of his parents. In brief, the blood genomic DNA of the patient and his parents was obtained using a blood genomic extraction kit (DP304, Tiangen). Efficient amplification primers (F AGCTGGTCAAGGCGACACTGG and R TCCTCTTGTGTGCTCTGGGCT) were designed near the *SYCE1* mutation site, and the specificity of the primers was verified by BLAST search of the NCBI genome database. The annealing temperature of primers was set at 60°C. The high‐fidelity enzyme (AS221, TransGen Biotech) was used to amplify the target sequence. The amplified products were separated by 2% agarose gel electrophoresis, and the target band was collected for Sanger sequencing. Finally, the sequencing results were compared with the *SYCE1* reference sequence and analysed to identify the mutation site.

### 2.5 IHC

The testis tissue of the NOA patient was obtained during the microscopic sperm extraction operation, and the testis tissue of the OA control patient was obtained by puncture. Paraffin embedding and routine immunohistochemical staining were performed after fixation with 4% paraformaldehyde (PFA). Briefly, paraffin‐embedded samples were sliced, baked and dewaxed, and then antigen repair with microwaves and blocking with serum were conducted. Next, these 3 μm slices were incubated with the SYCE1 primary antibody (17406, Proteintech, Wuhan, China) diluted 1:200 for 15 h in a 4°C damp environment. After several rinses with phosphate‐buffered saline (PBS), they were incubated with HRP‐labelled goat anti‐rabbit secondary antibody (A0208, Beyotime Biotechnology) at 37°C for 30 min. After washing, DAB was used to develop the colour. The nuclei were stained blue with haematoxylin for approximately 2 min. The brown sites indicated positive SYCE1 expression. Finally, the dehydrated and sealed slices were observed and captured using an Olympus BX53 biological microscope.

### 2.6 Western blot assay

Western blot assay was used to detect the change in SYCE1 expression in the patient and SYCE1 overexpression in the 293T cell line. Approximately 2 ml of peripheral blood was centrifuged to collect blood cell pellets. In order to collect the cell clumps, 293T cells were trypsinized (0.5%) and centrifuged. After washing with PBS, the cells were lysed with protease inhibitor‐containing RIPA lysis buffer (P0013B, Beyotime Biotechnology). Then, SDS was added to the cell samples, and proteins were denatured by high‐temperature boiling. The primary antibody used was the same as that used for IHC and diluted 1:1000. After incubation with HRP‐labelled secondary antibody, the target band was exposed to an ultra‐sensitive ECL reagent (P0018, Beyotime Biotechnology). GAPDH (ab8245, Abcam) was used as the internal control and diluted 1:1000.

All protein bands were quantified as IntDen values using the ImageJ software (V1.4.3, National Institute of Health). The relative expression of SYCE1 in each sample was determined by normalization to GAPDH. The statistical bar shows the fold change of SYCE1 in the test group relative to the control group, which is indicated as the mean ± SD. Differences between the two groups were counted by two independent samples t test, and significance was set at *p *< 0.05.

### 2.7 Plasmid construction for wild‐ and mutated‐type *SYCE1*

Normal testis tissue was obtained by the puncture method from an OA patient. RNA was extracted using the TRIzol method, and reverse transcription was performed to produce total cDNA (AT341, TransGen Biotech). The full‐length coding sequence of the wild‐type *SYCE1* variant 3 was produced by PCR amplification. The primer pair containing the restriction enzyme sites was F ggaattccATGGCGGGGAGGTCCCTGAC and R gcgtcgacACTCCACCATGTCACTGGTC. The annealing temperature of primers was set at 63°C. The mutant *SYCE1* sequence was prepared using overlap extension PCR. The primer pair covering the mutation site was ATGAGGGACTCTCTCCGCAGCCAG and CTGGCTGCGGAGAGAGTCCCTCAT. Both wild‐type and mutant coding sequences were ligated to the EGFP‐N1 overexpression vector by restriction enzyme digestion and ligation. Thus, SYCE1 was fused with the EGFP tag for expression in vitro.

### 2.8 Transfection of *SYCE1* plasmids into 293T cell line

The 293T cell line was cultured in DF12 medium (11320082, Gibco) supplemented with 10% foetal bovine serum. The cells proliferated to 70% confluence prior to transfection. The constructed wild‐ and mutant‐type *SYCE1* plasmids were extracted using an endotoxin‐removing kit (DP118, Tiangen), and cell transfection was carried out according to the protocol of lip2000 (11668500, Invitrogen). In brief, the total amount of plasmid added to cells in a 60 mm culture dish was approximately 5 μg. After 24 h of transfection, the plasmid expression was observed under a Nikon fluorescence microscope at 100× magnification. After 72 h, the cells were collected to examine SYCE1 expression according to standard Western blotting procedures.

## 3 RESULTS

### 3.1 Human subject

**FIGURE 1:**
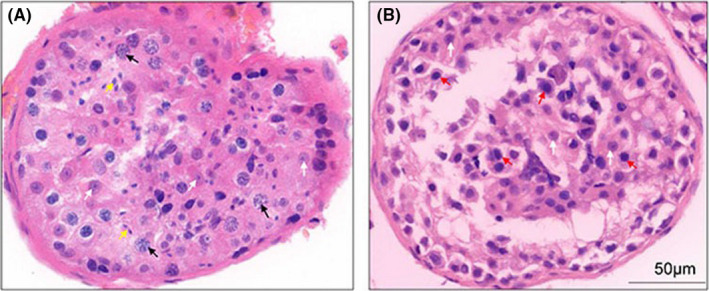
Pathological haematoxylin and eosin staining of testicular tissues from an OA control and the NOA patient. (A) The testicular puncture tissue of an OA control with normal spermatogenesis. The black arrows point to the primary spermatocytes in the pachytene phase, and the yellow arrows point to the mature sperm cells. (B) The testicular tissue of the NOA patient. The red arrows point to the blocked primary spermatocyte without mature forms. White arrowheads indicate Sertoli cells both in OA and NOA samples. NOA, non‐obstructive azoospermia (NOA); OA, obstructive azoospermia

The patient in this study was 29 years old, and there was no history of pregnancy following years of attempted conception. Information provided by the patient indicated that his parents were not close relatives. The patient has a brother and a sister who were able to naturally conceive a daughter and a son respectively. After centrifugation of the patient's semen, no sperm were found in the microscopic examination. Haematoxylin and eosin staining of the testicular tissue obtained during the microscopic sperm extraction operation showed that there was no mature sperm production in the seminiferous tubules, and the spermatocyte chromosome distribution was abnormal (Figure 1). Based on the above results, the patient was diagnosed with NOA.

### 3.2 High‐throughput sequencing identified a novel homozygous variant in *SYCE1*

**FIGURE 2:**
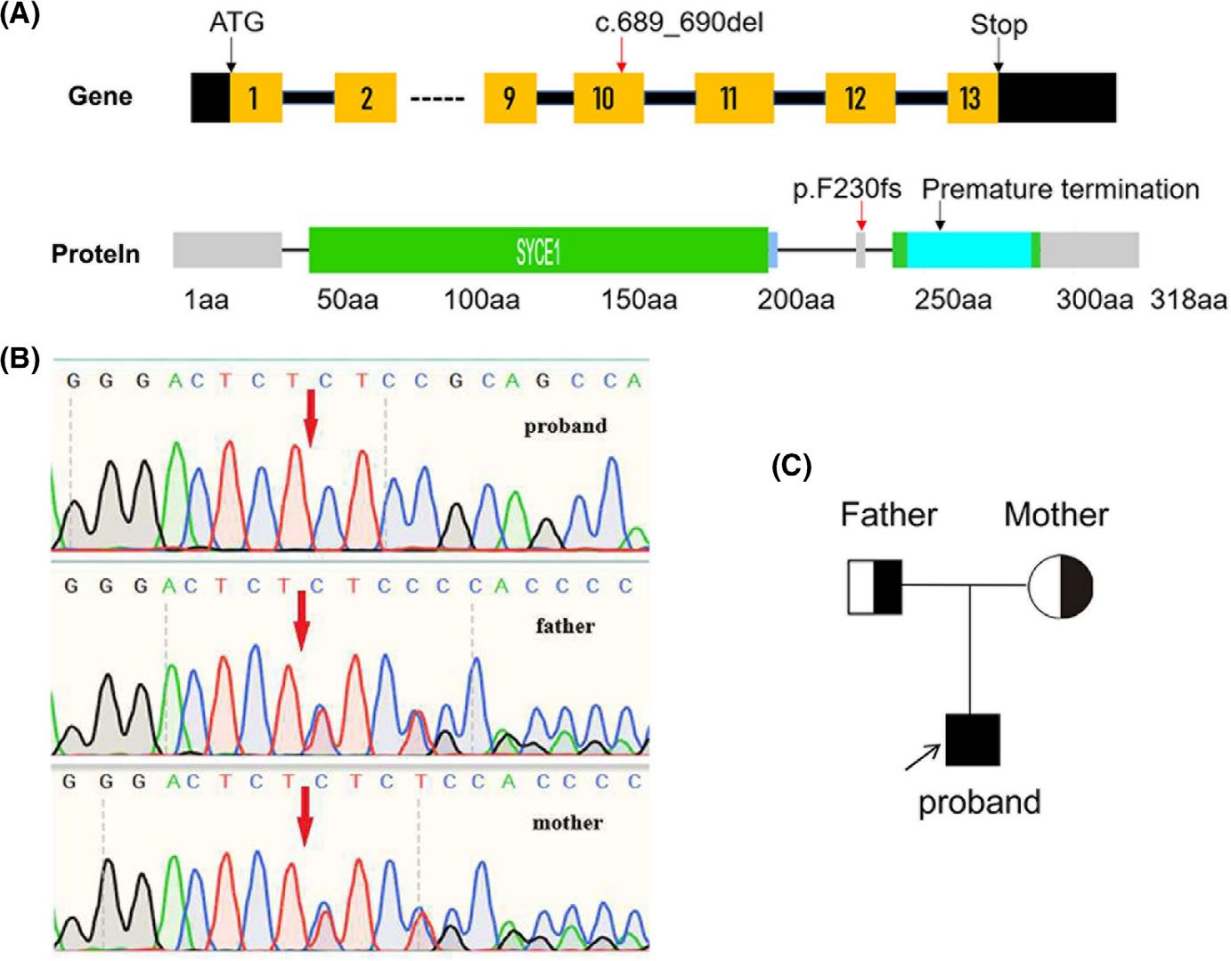
Identification of a novel exon mutation in the *SYCE1* gene of the NOA patient and his parents. (A) High‐throughput sequencing revealed a homozygous deletion mutation in the *SYCE1* tenth exon, which leaded to premature termination of translation. Gene structure: exons, noncoding exons, intron, ‐‐‐‐omitted exons 3 to 8. Protein domains: disorder, coiled‐coil, low_complexity. Red characters indicate the mutant in the *SYCE1* of the patient. (B) Sanger sequencing confirmed the homozygous mutation of *SYCE1* (c.689_690del; p.F230fs) in the patient and heterozygous mutations among his parents. (C) The pedigree of the NOA patient is shown

To explore possible infertility‐related gene mutations in the patient, we used target region capture combined with high‐throughput sequencing to detect the patient's peripheral blood DNA. As a result, the average sequencing depth of the target area was 320.73× and reached 99.39% coverage, meeting the screening requirements. A homozygous mutation (c.689_690del; p.F230fs) was found in the *SYCE1* gene of the patient, with the tenth exon missing TT bases (Figure 2A). The frequencies in the ExAC database and 1000 human genomes were considerably low. The other tested male infertility‐related genes were normal. The identified homozygous mutation was verified by Sanger sequencing. We also detected a *SYCE1* mutation in his parents by Sanger sequencing and found that they both showed single allelic mutations at the same site (Figure 2B). Thus, this novel *SYCE1* mutation was inherited recessively (Figure 2C) and was consistent with family co‐segregation. Mutation Taster revealed that this SYCE1 mutation (c.689_690del; p.F230fs) was pathogenic (0.999) (Table 1). The patient was clinically diagnosed with azoospermia. Therefore, the variation was considered to be pathogenic variation according to the interpretation standards and guiding principles of gene variation issued by the ACMG in 2015.

**TABLE 1:** In silico analysis of this novel *SYCE1* variant

Mutation	Amino acid change	Mutation Taster	Variation viewer	CO‐ESP	ExAC (total)	ExAC East Asian	1000 G MAF
c.689_690del	p.F230fs	Disease causing (0.999)	no report	0	0	0	0

### 3.3 Expression and localization of mutated SYCE1 in the NOA patient

**FIGURE 3:**
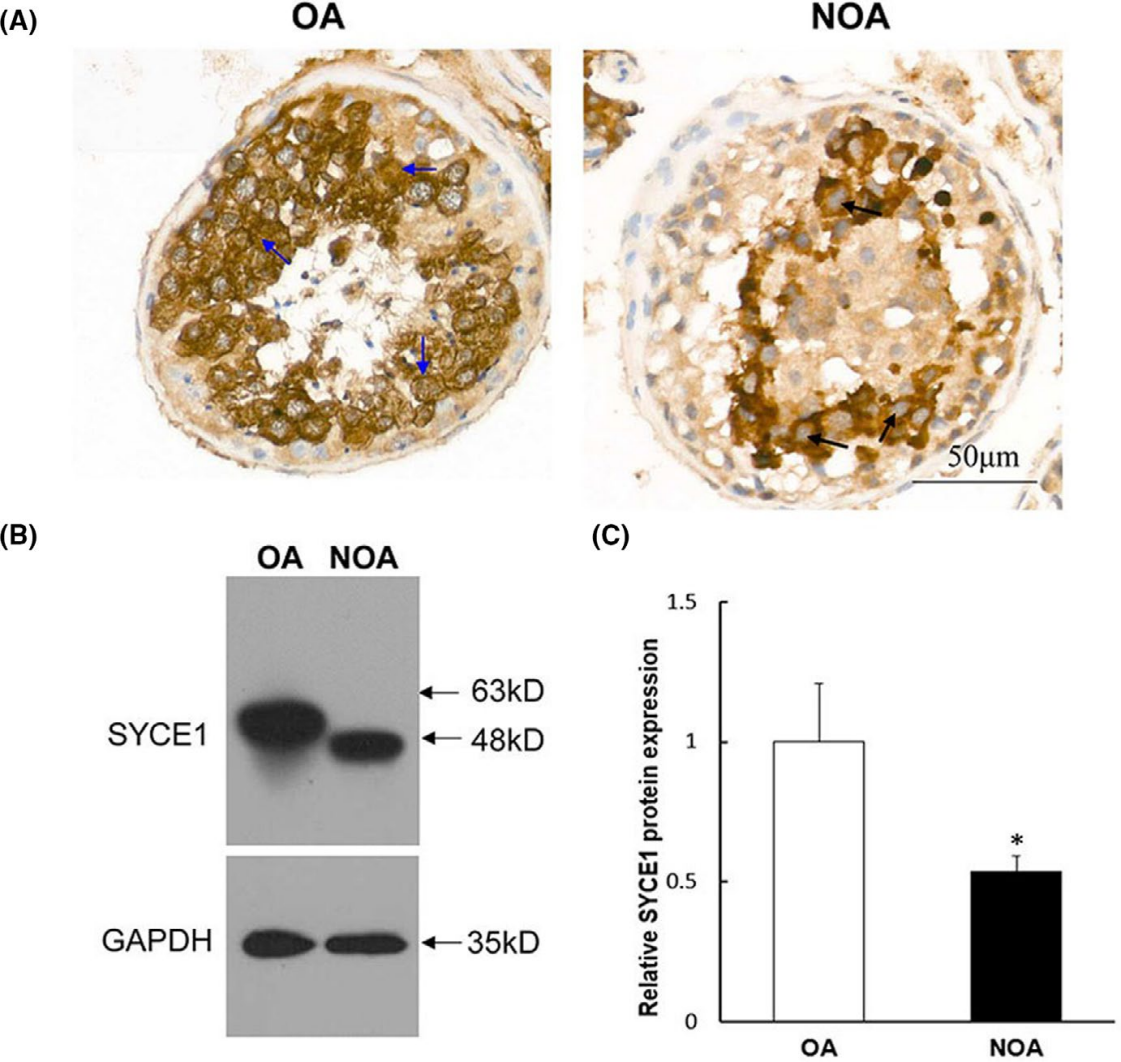
Examination of mutated SYCE1 protein expression and localization in the NOA patient. (A) Immunohistochemical staining showed the endogenous expression and localization of wild‐ and mutated‐type SYCE1. The blue arrows indicated that SYCE1 was expressed in the nucleus of spermatocytes of the OA control. The black arrows indicated that SYCE1 was undetected in the nucleus of arrested spermatocytes of the NOA patient. (B) Western blotting (WB) was used to study the effect of SYCE1 mutation on its endogenous protein expression. The total protein was extracted from the whole blood cells, and GAPDH was selected as the internal control of WB. (C) Grey values (IntDen) of WB bands in (B) were calculated, and the relative protein expression difference between groups was counted. ‘*’ represented statistically significant difference. NOA, non‐obstructive azoospermia; OA, obstructive azoospermia

Sequence analysis indicated that c.689_690del can cause a frameshift mutation in SYCE1 and lead to premature termination of translation. According to our calculation, 69 amino acids may be missing for SYCE1 in the NOA patient compared with wild‐type SYCE1. To explore the effects of a homozygous mutation (c.689_690del; p.F230fs) on SYCE1 endogenous expression, the patient's blood cells were used to detect endogenous SYCE1 expression by Western blotting. As a result, SYCE1 protein size was significantly reduced compared to that in the wild‐type (Figure 3B). We further examined the distribution of the mutated SYCE1 protein in the testis tissue using immunohistochemistry (IHC). The results showed that the nuclear localization of SYCE1 was abnormal in the NOA patient, migrating from the nucleus to the cytoplasm (Figure 3A).

### 3.4 Effects of SYCE1 mutation on its expression and localization in vitro

**FIGURE 4:**
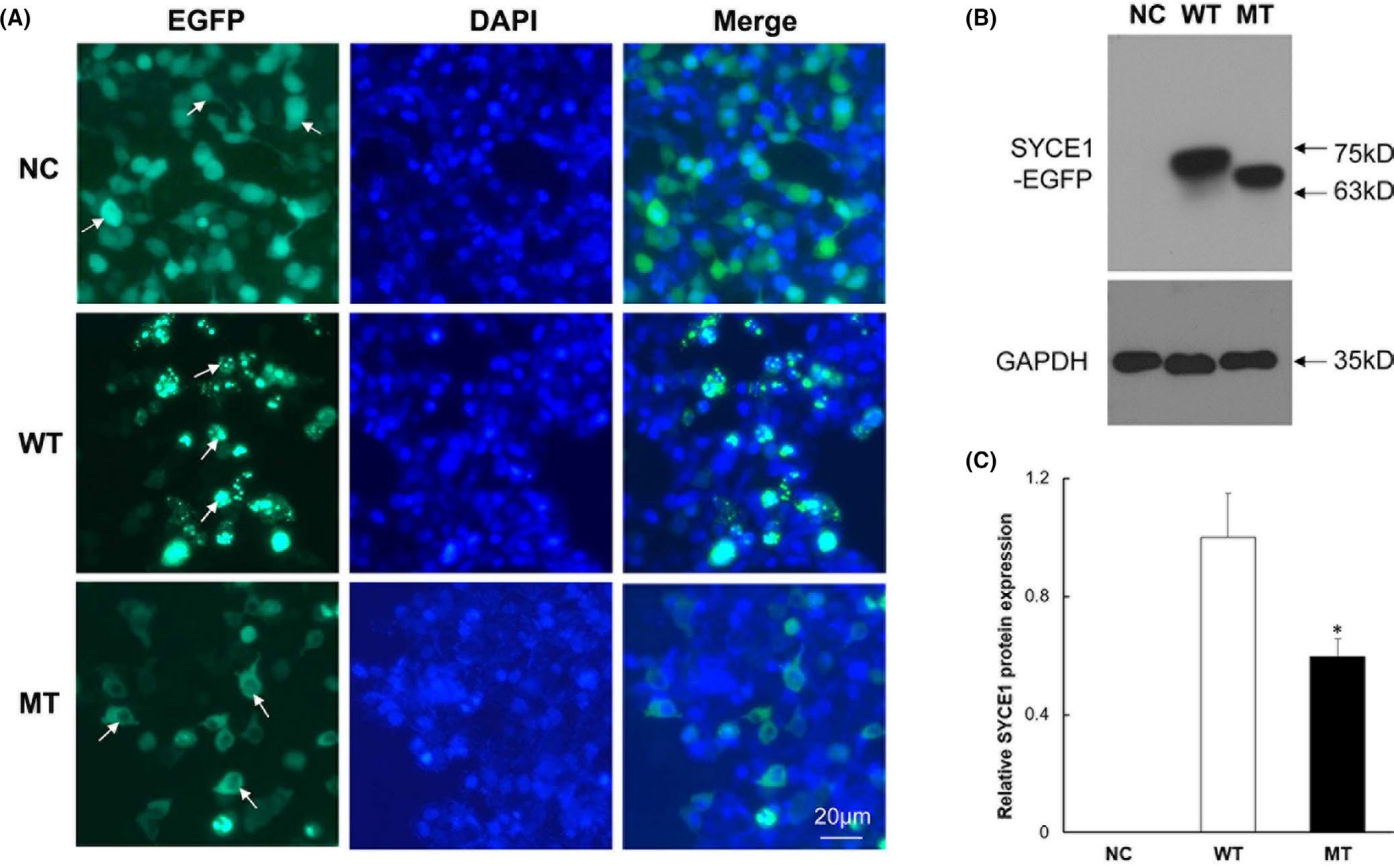
Observation of wild‐ and mutated‐type SYCE1 expression in vitro. (A) Wild‐type and mutant SYCE1 plasmids were separately transfected into the 293T cell line to observe the nuclear localization of corresponding proteins. Green EGFP fluorescence indicated exogenous SYCE1 sites. DAPI stained all cell nuclei blue. (B) WB assay detected the expression and size of mutant SYCE1 protein. The protein molecular weight represents the fusion expression of SYCE1 and EGFP. (C) Grey values (IntDen) of WB bands in (B) were calculated, and the relative protein expression difference between groups was counted. ‘*’ indicated statistically significant difference (*p *< 0.05). NC represents the empty plasmid containing an EGFP tag; WT represents the wild‐type SYCE1 plasmid, and MT represents the mutant SYCE1 plasmid that mimics the NOA patient. EGFP, enhanced green fluorescent protein; MT, cells transfected with mutated‐SYCE1 plasmid containing the c.689_690del; NC, cells transfected with empty plasmid containing an EGFP tag; WT, cells transfected with wild‐SYCE1 plasmid

To demonstrate the effects of the SYCE1 homozygous mutation on its protein expression and localization in vitro, we constructed wild‐SYCE1 and mutated‐type SYCE1 (c.689_690del; p.F230fs) expression vectors and transfected them into the human 293T cell line. Following 24 h of transfection, the fusion expressed EGFP fluorescence, indicating that the SYCE1 mutation affected its localization, causing it to transfer from the nucleus to the cytoplasm (Figure 4A). Wild‐type SYCE1 clustered in the nucleus, whereas mutated‐type SYCE1 was diffusely distributed in the cytoplasm. After 72 h of transfection, we collected different groups of cells and performed Western blotting. The results showed that the expression of mutant SYCE1 was significantly decreased compared to that of the wild type (Figure 4B). In addition, the molecular weight of the mutated‐type SYCE1 was remarkably reduced, which was consistent with the endogenous mutation in the NOA patient.

## 4 DISCUSSION

The SC is located between homologous chromosomes during the first meiosis,^26^ and SYCE1 is an important component of the SC, located in the central region.^27^ Recently, two reports have shown that the *SYCE1* gene mutation is related to male NOA. They occurred in two consanguinity families in the Middle East, in which the mutation sites were located in the intron region and may have affected alternative splicing.^5^, ^19^ Another case was a nonsense homozygous mutation leading to POI, which occurred in two sisters in an Israeli Arab family.^18^ This study reported, for the first time, a case of a frameshift mutation (c.689_690del; p.F230fs) in the exon region of the *SYCE1* gene in a Chinese NOA patient. Cellular and molecular studies on this new homozygous mutation were carried out to clarify its effect on SYCE1 protein expression.

Pathological analysis showed that there was no mature sperm production in the testicular tissue of the NOA patient, and the spermatocytes had abnormal meiosis. Screening of male infertility‐related genes revealed a new homozygous mutation (c.689_690del; p.F230fs) in the exon region of the *SYCE1* gene in the patient. Both parents of the patient were identified as having *SYCE1* monoallelic mutations. The novel *SYCE1* mutation was in line with recessive inheritance in the family, consistent with previous reports.^5^, ^19^ The testes of mice lacking *SYCE1* have been reported to contain a large number of primary spermatocytes; however, the meiotic maturation process is blocked.^20^ In this NOA patient with the *SYCE1* mutation (c.689_690del; p.F230fs), we observed similar pathological results as the above report.

To explore the effects of c.689_690del on SYCE1 protein expression, we first detected the endogenous SYCE1 expression change in the patient and then constructed the mutant *SYCE1* vector for in vitro experimental verification. The testicular tissue sample obtained from the puncture was small and had been fixed with PFA; therefore, the peripheral blood of the patient was used to examine the endogenous expression of the mutated SYCE1 protein. In both the patient and in vitro experiments, we found that SYCE1 expression was decreased. Hence, we discovered that the new homozygous mutation (c.689_690del; p.F230fs) did not cause the complete loss of SYCE1 expression; however, it might have increased *SYCE1* mRNA instability or affected its translation efficiency. This was different from the impact of the known c.197‐2A> G mutation.^5^ Additionally, our studies showed that the molecular weight of the mutant SYCE1 protein decreased. It is believed that the premature termination of translation caused by the frameshift mutation was responsible for this, according to the mutation sequence analysis. Notably, the immunohistochemical study suggested that the nuclear localization of the mutated SYCE1 was abnormal in the patient's testicular tissue, which was confirmed by in vitro cell research. The mutant SYCE1 (c.689_690del; p.F230fs) failed to locate in the nucleus, unlike the wild‐type one. EGFP fluorescence from an empty plasmid was distributed both in the nucleus and in the cytoplasm, because EGFP protein size is small and can enter the nucleus freely.

SYCE1 is an important component of the SC.^21^ In this study, we demonstrated that the new SYCE1 mutation (c.689_690del; p.F230fs) led to abnormal subcellular localization. Thus, it should have a significant impact on the formation of SCs during meiosis and hinder normal meiosis and gamete maturation. This new SYCE1 mutation caused the failure of its nuclear localization, possibly because it affected its nuclear localization signal. However, analysis of the NLS Mapper online software revealed that the SYCE1 mutation (c.689_690del; p.F230fs) was not located in the predicted nuclear localization signals (data not presented). Previous studies have shown that the recruitment of SYCE1 to the SC depends on SYCP1, SYCE3 and SIX6OS1, and the C‐terminus of SYCE1 interact with SYCE3.^22^, ^23^, ^24^, ^25^ Sequence analysis revealed that the novel SYCE1 mutation (c.689_690del; p.F230fs) destroys the C‐terminal amino acid sequence structure. Therefore, it is speculated that the mutation may affect the binding of SYCE1 to its interacting proteins and interfere with its nuclear localization and molecular function. The underlying molecular mechanisms need to be studied in depth in the future.

This study identified a new type of pathogenic homozygous mutation (c.689_690del; p.F230fs) in the exon region of *SYCE1*. We conducted cellular and molecular studies to clarify the effects of the new mutation on the expression and localization of the SYCE1 protein. This study is beneficial in understanding the molecular basis of abnormal gamete meiosis and the pathogenesis of NOA, and provides a theoretical basis for the development of potential gene therapy strategies.

## CONFLICT OF INTEREST

The authors conform that there are no conflicts of interest.

## AUTHOR CONTRIBUTIONS

**Ke Feng:** Conceptualization (equal). **Hengtao Ge:** Project administration (equal); Resources (equal). **Huanhuan Chen:** Data curation (equal); Methodology (equal). **Chenchen Cui:** Formal analysis (equal); Software (equal); Visualization (equal). **Shan Zhang:** Investigation (equal). **Cuilian Zhang:** Writing – review & editing (equal). **Li Meng:** Writing – review & editing (equal). **Haibin Guo:** Supervision (equal); Validation (equal). **Lei Zhang:** Funding acquisition (equal); Writing – original draft (equal).

## Supporting information

Table S1

## Data Availability

The data that support the findings are available from the corresponding author upon reasonable request.
