# Supplementary material for: Novel exon mutation in SYCE1 gene is associated with non‐obstructive azoospermia
Source: J Cell Mol Med. 2022 Jan 12;26(4):1245–52. doi: 10.1111/jcmm.17180 (PMC8831938; doi:10.1111/jcmm.17180)
Supplement: Supplementary file 1 — Table S1 [file JCMM-26-1245-s001.docx]

**Supplementary table 1.** The male infertility-related gene set covered by the target area capture combined with the high-throughput sequencing used in this study.

| **Categories** | **Gene numbers** | **Gene names** |
| --- | --- | --- |
| Spermogenesis disorders | 16 | *MEIOB NANOS1 ETV5 RHOXF1 PLCZ1 SOHLH2 SYCE1 SYCP3 RHOXF1 RHOXF2 HSF2 USP26 TEX11* |
| Abnormal sperm morphology and movement | 23 | *AURKC DNAH1 PRM1 HYDIN DYX1C1 SEPT12 SPAG17 ZMYND10 HEATR2 CCDC39 DNAAF3 DNAAF2 DNAAF1 CATSPER1 SPAT16 RSPH9 SUN5 DNAL1 DNAI2 DNAI1 DNAH5 DNAH11 DPY19L2* |
| Idiopathic hypogonadotropic hypogonadism | 14 | *KAL1 CHD7 LEP LEPR PROK2 PROKR2 FGFR1 FGF8 KISS1R NELF GNRHR TAC3 WDR11 TACR3* |
| Abnormal sexual development | 2 | *AR NR5A1* |
| Congenital absence of vas deferens | 1 | *CFTR* |
